# The Terry Fox Research Institute Canadian Prostate Cancer Biomarker Network: an analysis of a pan-Canadian multi-center cohort for biomarker validation

**DOI:** 10.1186/s12894-018-0392-x

**Published:** 2018-09-10

**Authors:** Véronique Ouellet, Armen Aprikian, Alain Bergeron, Fadi Brimo, Robert G. Bristow, Simone Chevalier, Darrel Drachenberg, Ladan Fazli, Neil E. Fleshner, Martin Gleave, Pierre Karakiewicz, Laurence Klotz, Louis Lacombe, Jean-Baptiste Lattouf, Theodorus van der Kwast, Jeremy A. Squire, Mathieu Latour, Dominique Trudel, Anne-Marie Mes-Masson, Fred Saad

**Affiliations:** 1 Institut du cancer de Montréal and Centre de recherche du Centre hospitalier de l’Université de Montréal, 900, St-Denis St, room R10-464, Montréal, Québec H2X 0A9 Canada; 2 Research Institute of McGill University Health Center and Department of Surgery (Urology), McGill University, Montréal, Québec Canada; 3 CHU de Québec-Université Laval and Department of Surgery, Université Laval, Québec City, Québec Canada; 4 Department of Pathology, McGill University Health Centre, Montréal, Québec Canada; 5 Department of Medical Biophysics and Department of Radiation Oncology, University of Toronto, Toronto, ON Canada; 6 University Health Network, Toronto, ON Canada; 7 University of Manitoba and Manitoba Prostate Centre, Winnipeg, MB Canada; 8 Vancouver Prostate Centre, Vancouver, BC Canada; 9 Division of Urology, Department of Surgery of University Health Network, University of Toronto, Toronto, ON Canada; 10 Department of Urologic Sciences, Vancouver, BC Canada; 11 Cancer Prognostics and Health Outcomes Unit, Centre hospitalier de l’Université de Montréal, Montréal, Québec Canada; 12 Department of Surgery, Université de Montréal, Montréal, Québec Canada; 13 Sunnybrook Health Sciences Centre, Toronto, ON Canada; 14 Department of Pathology and Molecular Medicine, Queen’s University, Kingston, ON Canada; 15 Department of Pathology and Cellular Biology, Université de Montréal, Montréal, Québec Canada; 16 Department of Medicine, Université de Montréal, Montréal, Québec Canada; 17 Department of Genetics and Pathology, Ribeirão Preto Medical School, University of São Paulo, Ribeirão Preto, Brazil

**Keywords:** Prostate cancer, Tissue microarray, Biomarker validation, Immunohistochemistry, Patient prognosis

## Abstract

**Background:**

Refinement of parameters defining prostate cancer (PC) prognosis are urgently needed to identify patients with indolent versus aggressive disease. The Canadian Prostate Cancer Biomaker Network (CPCBN) consists of researchers from four Canadian provinces to create a validation cohort to address issues dealing with PC diagnosis and management.

**Methods:**

A total of 1512 radical prostatectomy (RP) specimens from five different biorepositories affiliated with teaching hospitals were selected to constitute the cohort. Tumoral and adjacent benign tissues were arrayed on tissue microarrays (TMAs). A patient clinical database was developed and includes data on diagnosis, treatment and clinical outcome.

**Results:**

Mean age at diagnosis of patients in the cohort was 61 years. Of these patients, 31% had a low grade (≤6) Gleason score (GS), 55% had GS 7 (40% of 3 + 4 and 15% of 4 + 3) and 14% had high GS (≥8) PC. The median follow-up of the cohort was 113 months. A total of 34% had a biochemical relapse, 4% developed bone metastasis and 3% of patients died from PC while 9% died of other causes. Pathological review of the TMAs confirmed the presence of tumor and benign tissue cores for > 94% of patients. Immunohistochemistry and FISH analyses, performed on a small set of specimens, showed high quality results and no biorepository-specific bias.

**Conclusions:**

The CPCBN RP cohort is representative of real world PC disease observed in the Canadian population. The frequency of biochemical relapse and bone metastasis as events allows for a precise assessment of the prognostic value of biomarkers. This resource is available, in a step-wise manner, for researchers who intend to validate prognostic biomarkers in PC. Combining multiple biomarkers with clinical and pathologic parameters that are predictive of outcome will aid in clinical decision-making for patients treated for PC.

**Electronic supplementary material:**

The online version of this article (10.1186/s12894-018-0392-x) contains supplementary material, which is available to authorized users.

## Background

The inability to clearly distinguish indolent versus aggressive disease is a major challenge for physicians caring for patients with prostate cancer (PC) [1]. Patients are stratified into groups ranging from very low to very high risk based on prostate-specific antigen (PSA) levels at time of diagnosis, tumor Gleason score (GS) in biopsies, and tumor stage at clinical presentation [2–4]. However, the biology of PC reflects a multifocal and multiclonal nature of tumors that is far more complex than initial predictions from current clinical parameters [5–7].

Foremost, the prognostic ability of new biomarkers should determine the risk for lethal PC and track disease progression in order for the therapy to be modified [2]. Several emerging biomarker candidates have been described; none so far have been fully validated or robust enough to be added to clinical parameters used in practice. Tissue microarrays (TMAs) represent a high-throughput platform to apply protein- and nucleotide-based assays enabling biomarker testing within a single tumor core [3] of hundreds of patient samples simultaneously [8]. Current strategies aspire to multiplex approaches combining current clinical parameters with a comprehensive panel of biomarkers to improve diagnostic accuracy of disease status and resolve the heterogeneity that confounds risk stratification in PC [2].

The Canadian Prostate Cancer Biomarker Network (CPCBN) represents a community of clinicians and researchers that is committed to improving the clinical management of PC. The CPCBN initiative is a validation rather than discovery platform and invites biomarker proposals from all researchers with preliminary evidence demonstrating their utility in PC management. An application to access the TMA platform is available on the CPCBN website, along with details on the CPCBN program and affiliated partners http://www.tfri.ca/en/research/translational-research/cpcbn.aspx). A compilation of potential biomarkers has already been reviewed and studies are underway using the radical prostatectomy (RP) cohort. Data for well-known PC biomarkers such as ERG, PTEN, Ki67 and AR will be available to researchers upon request. This platform serves as an invaluable resource for the entire PC research community, accelerating breakthroughs in PC research, and supporting the establishment of nomograms to predict patient progression.

In this study, we report a TMA-based validation process which includes assembly of a retrospective multi-center RP cohort to build TMAs that will evaluate both biomarkers and their utility in identifying patients at high risk for biochemical recurrence (BCR) and the development of metastases or PC-specific mortality. To ensure homogeneity across sites, we used the Canadian Tumor Repository Network (CTRNet, www.ctrnet.ca) standards for quality assurance and developed standard operating procedures (SOPs). We also report on the quality control of this RP TMA series with quality assessments and controls, focusing on the TMA suitability for immunohistochemistry (IHC) and fluorescence in situ hybridization (FISH) techniques.

## Methods

### Patient cohort and participating centers

RP specimens were selected from five different biobanks affiliated with academic health care centers across Canada: Centre hospitalier de l’Université de Montréal (CHUM), CHU de Québec-Université Laval (CHUdeQ-UL), McGill University Health Centre (MUHC), Vancouver Prostate Centre (VPC), and University Health Network (UHN). The selected specimens were biobanked between 1990 and 2011. All patients signed an informed consent to participate within one of the above listed biobanks and agreed to the use of their specimens and data for research purposes. Inclusion criteria included: RP specimens archived as formalin-fixed paraffin-embedded (FFPE) blocks, treatment (hormone or chemotherapy) naïve patients with a minimum follow-up of 24 months. Patients with severe comorbidity were naturally excluded as they are not candidates for RP surgery. Each center received ethical approval from their Institutional Review Board (IRB) for biobanking activity and for their contributions to the CPCBN. CTRNet standards were followed for quality assurance and ensured appropriate handling of human tissue.

### Clinical data management

Clinical data for each patient were collated into an Advanced Tissue Management (ATiM) database developed by the CTRNet and customized for the CPCBN. Complete clinical data provided the month and year of diagnosis and surgery, age, pretreatment PSA level, pathologic stage, Gleason grade, margin status, date of BCR, PSA progression, development of metastasis, and treatments received following RP when applicable. BCR endpoints were based on serum PSA measurement in three different conditions: PSA levels of 0.2 ng/mL and rising, a PSA level followed by salvage/adjuvant treatment and finally, when initial post-operative PSA levels were greater than 0.2 ng/mL and rising following surgery (failed RP). Appearance of bone metastasis and PC mortality were considered ultimate endpoints.

### TMA construction

TMA construction was performed at each site: CHUM, UHN, and VPC used the TMArrayer (Pathology Devices, Inc., Westminster, MD, USA), while the CHUdeQ-UL and MUHC used the manual tissue arrayer, MTA-1 (Beecher Instruments, WI, USA). A pathologist selected the FFPE block, and the area of interest (tumor or adjacent benign) was circled directly onto the hematoxylin and eosin (H&E) stained slide. Cores of 0.6 mm were extracted from the corresponding FFPE block and arrayed on a receiver paraffin block. A SOP was developed and guided the construction of the different TMA series.

### TMA design

To build the quality control TMA (QC-TMA), a TMA block was circulated across four sites where three tumor cores from 10 PC specimens were arrayed to evaluate the feasibility of the multi-center resource. Due to specific institution requirements, one site arrayed their specimens on a separate TMA, resulting in two QC-TMA blocks. However, sections were combined onto the same glass slide for subsequent analyses. The optimization TMA (OPT-TMA) was constructed at the CHUM and included banked tissues from 15 RP, 5 breast cancer and 5 ovarian cancer cases along with mouse xenograft tissues derived from human PC cell lines 22RV1, LNCaP, DU145 and PC3. The Test-TMA series was composed of 250 RP specimens selected from four biobanks: CHUM, MUHC, CHUdeQ-UL (50 RP specimens each), and VPC (100 RP specimens). Each TMA block contained three cores of tumor and two cores of adjacent benign tissues from 50 RP cases. The Validation-TMA series contain prostate tissues from 1262 specimens across five centers. Three to four cores of tumor and one to two cores of adjacent benign tissues were arrayed on receiver blocks. Validation-TMAs also contained 50 RP cases per block with a few exceptions. On each TMA block composing the test or the validation series, two cores of the 4 PC cell line-derived xenografts used for the OPT-TMA were also included. After a first pathology review, cores were repunched as necessary and resulted in a total of seven TMA blocks for the Test-TMA and 31 TMA blocks for the Validation-TMA series.

### IHC staining and analysis

Tissue quality was assessed with the following markers: PSMA, PSA, p63, P504s, P501s, Ki67, AR, CK18, and HMW-CK. Details about antibody sources, dilutions, antigen retrieval and incubation conditions are described in Additional file 1. QC-TMA slides were stained at the coordinating center (CHUM) using the BenchMark XT automated stainer (Ventana Medical System Inc.). TMA slides were scanned and assessed visually for analysis (OlyVIA, Olympus, ON, Canada). Two independent observers blindly scored the percentage of stained cells for all markers except for PSA and CK18 where the intensity of staining was also evaluated.

### FISH analysis

The *PTEN* FISH probe consisting in a four-color probe combination detecting *PTEN*, *WAPAL, FAS* and *CEP 10*, was obtained from CymoGenDx/Biocare Medical (Concord, CA) and was used as previously described [9]. The pathologist selected areas of TMA sections stained with DAPI, which were analyzed against immediately adjacent sections stained with H&E. *PTEN* copy number was determined by counting signals of all four markers in 50–100 distinct and intact interphase nuclei per tumor core using SemRock filters selected for excitation/emission spectra of each probe. Cores that showed visible deletions were scored by reviewing 50 cells per core. Hemizygous (single copy) *PTEN* deletion denoted cores with 50% of nuclei exhibiting clonal loss of *PTEN* whereas homozygous *PTEN* deletion was assigned to cores with loss of both *PTEN* loci in 30% of nuclei.

### Central pathology review

Central pathology review assessed RP specimens of the QC-TMA, Test-TMA and Validation-TMA series. Scoring criteria included GS, the amount of glandular tissue present, and specificity of the core nature in terms of adjacent benign, cancer, prostatic intraepithelial neoplasia (PIN), intraductal carcinoma (IDC), atypical small acinar proliferation (ASAP), stroma, muscle or inflammation [10]. Upon review, cores were qualified as informative if the specific tissue of interest (adjacent benign or cancer) was present in at least 5 to 10% of the core area. Additional cores were requested for replacement if less than two cores for either tumor or adjacent benign tissues did not meet established criteria. Replacement cores were reviewed and added to a new or existing array, and if tissue samples were depleted, additional cores or patients were included to complete the full cohort.

### Statistical analyses

Hierarchical clustering analysis of markers assessed in the QC-TMA was performed with Genespring software (Agilent Genomics, CA, USA) using Pearson correlation as a similarity measure and an average linkage-clustering algorithm. Survival analyses (Cox regression and Kaplan-Meier curves) were performed using the IBM SPSS Statistics (Version 23) software.

## Results

### Feasibility of the multi-center TMA-based resource

**Fig. 1:**
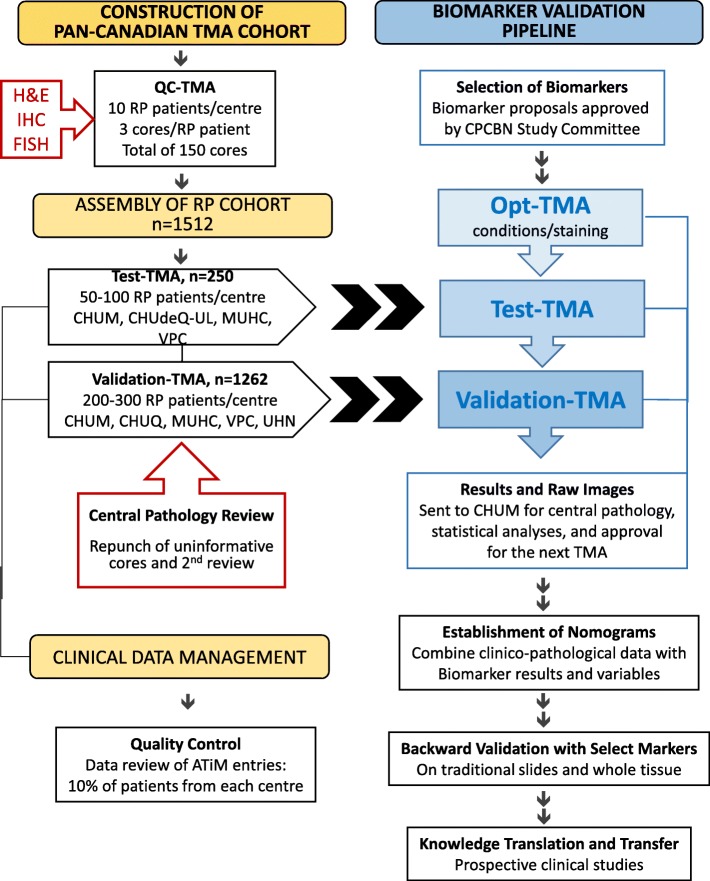
Design of the CPCBN Validation Tissue Microarray Platform for Prostate Cancer Biomarkers

**Fig. 2:**
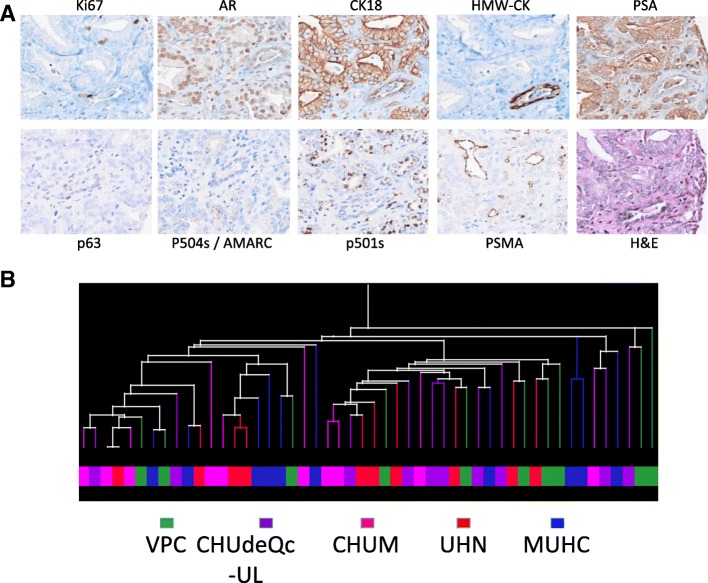
Immunohistochemistry and hierarchal clustering analysis of biobanked specimens arrayed in the QC-TMA, representing 50 radical prostatectomy cases from five different centres (total of 150 cores). **a** IHC evaluation with nine protein tissue markers. **b** Hierarchal clustering based on IHC detection of the nine different markers in samples of different center origin, corresponding to the colour legend below

A quality control TMA (QC-TMA) was constructed (Fig. 1, left column) using three cores from 10 PC specimens with GS 7 from each of the five biorepositories. The 150 cores composing this array were evaluated for tissue integrity, antigenicity, and performance in protein and nucleic acid-based assays. These cores were tumors of expected Gleason grade (in at least 2/3 cores) in 94% (47/50) of samples. Tissue integrity and antigenicity was determined by evaluation of the expression of nine different markers with nuclear and/or cytoplasmic localization (Ki67, AR, CK18) in addition to markers that distinguish tumor vs. benign glands (HMW-CK, p63, and P504S/AMACR) or proteins usually expressed by prostate cells (PSA, P501S and PSMA) (Fig. 2a). Hierarchal clustering using a Pearson centered distance metric was based on the detection of these nine markers. Hierarchal clustering demonstrated that there was no site-specific bias (Fig. 2b).

**Fig. 3:**
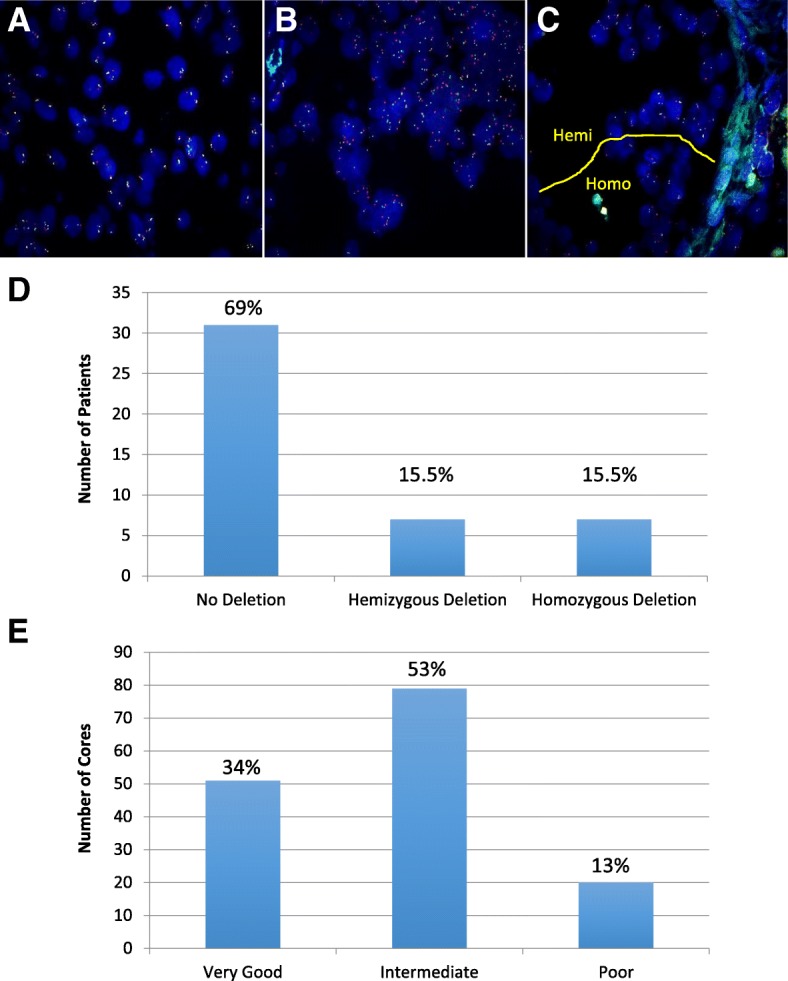
Fluorescence in-situ hybridization of the QC-TMA, with DNA probes detecting *PTEN* (orange), *WAPAL* (green), *FAS* (aqua), and *CEP* 10 (red). **a** Cells representing no *PTEN* deletion. **b** Cells showing homozygous *PTEN* deletion with relative hemizygous loss of *WAPAL* and *FAS* signal. **c** Cells in the same gland showing homozygous (Homo) and hemizygous (Hemi) *PTEN* deletions. **d** *PTEN* deletion status among the 50 patients in the QC-TMA. **e** Overall quality assessment of 150 cores for FISH analysis. Intermediate quality was assigned to 53% of cores that had a detectable *PTEN* deletion status but also had high background to signal ratios or had areas that were over-digested. Very good quality was observed for 34% of cores that produced strong signal over low background and even digestion throughout (Additional file 2)

Analysis of FISH data using the four-probe FISH assay, showed that *PTEN* deletions were hemizygous or homozygous at 15.5% each, whereas the majority of cores displayed no *PTEN* deletions (69%) (Fig. 3a-d). The FISH results reflected the quality of the cores, reported as very good, intermediate or poor (Fig. 3e). Approximately 13% of cores were considered of poor quality because the core was either absent due to mechanical processing/sectioning, over-digested, or else, yielded a poor signal (Additional file 2). These results highlighted the need for potential modifications for TMA FISH protocols to optimize digestion and reduce background signal. Nonetheless, PTEN status was assessed for 87% of the cores. Overall, the QC-TMA demonstrated the feasibility of coordinating a large multi-institutional cohort with specimens of acceptable quality on which protein and DNA markers could be assessed.

### Design and strategy of a biomarker validation process

Under the pipeline scheme (Fig. 1, right column), a study committee comprised of pathologists, clinicians and researchers selects promising markers for access to TMA resources and clinical data according to specific criteria (Table 1). This is followed by sequential evaluation through the TMA series starting with the OPT-TMA, which confirms reproducibility and reliability of staining conditions and reagents on different tissues. Upon successful completion of this step, the Test-TMA (*n* = 250), which evaluates the biomarker strength within a small subset representing the RP cohort, is released to the investigator. Finally, after evaluation of the performance of the biomarker, the large Validation-TMA, which contains the remaining cases of the entire RP cohort (*n* = 1262) is released. Both the OPT-TMA and Test-TMA represent checkpoints that determine whether biomarkers can advance along the pipeline. In the end, raw data and images are compiled and transferred to the coordinating center (CHUM) for repository and central pathology review, and secured for future nomogram development. This nomogram, once validated, could be used in a clinical setting to discriminate patients that would need a more aggressive treatment compared to those with a favorable prognosis.

**Table 1:** Biomarker selection criteria and considerations

Interest of the biomarker based on extensive preliminary data	
Relevance to CPCBN objectives and clinical impact for prostate cancer	
Cohort size used to determine biomarker status	
Assay performed on paraffin-embedded tissue or TMA	
Staining quality and requirements that include the following: • Reliable staining against controls and background levels using an automated stainer • Antibody specificity validated by western blot or IHC/immunofluorescence with appropriate controls • Preferences towards monoclonal antibody use • Preferences towards digital image analysis	
Specific role in prostate cancer prognosis and supporting statistical data (BCR, development of metastasis, *p* value)	
Sufficient resources for biomarker analysis (proposed laboratory, supportive infrastructure, funding, and partners)	

### Test and validation TMA evaluation

Each center selected 300 RP cases to build the Test and Validation TMAs. A central pathologist reviewed each TMA block to ensure the high quality of the resource (Table 2). Core assessments for sufficient material and accurate tissue representation (cancer or adjacent benign) determined which patient samples required additional cores. At least 2 cores of tumor tissues were arrayed from a total of 1429 patients (95%) whereas 1047 patients (69%) had 3 cores or more (Table 3). For the benign adjacent tissue, at least 1 core was obtained for 1496 patients (99%) and 2 cores for 1212 patients (80%) (Table 3).

**Table 2:** Central pathology review of all tissue cores contributing to the Test- and Validation-TMA series

	Sites				
	CHUM	CHUdeQ-UL	MUHC	UHN	VPC
EXPECTED BENIGN CORES	623	545	627	773	691
Reviewed as Benign	597	436	485	673	511
Reviewed as Cancer	0	19	67	25	88
Reviewed as Uninformative^a^	26	90	75	75	92
EXPECTED TUMOR CORES	954	1269	953	1042	1135
Reviewed as Cancer	845	944	707	825	825
Reviewed as Benign	52	133	109	109	192
Reviewed as Uninformative^a^	57	192	137	108	118

^a^Prostatic intraepithelial neoplasia, intraductal carcinoma, atypical small acinar proliferation, < 5% tumor cells, stroma only, muscle or inflammation

**Table 3:** Number and nature of cores included in the Test- and Validation-TMA series after central pathology review

Sites	Number of Patients	Number of Tumor Cores per Patient					Number of Benign Adjacent Cores per Patient				
		0	1	2	3	> 4	0	1	2	3	> 4
CHUM	304	2	6	55	236	5	1	16	241	37	9
CHUdeQ-UL	301	1	9	65	98	128	4	130	104	36	27
MUHC	304	6	26	93	167	12	5	50	208	37	4
UHN	303	9	10	111	119	54	1	37	177	28	60
VPC	300	5	9	58	165	63	5	51	162	49	33
Total	1512	23	60	382	785	262	16	284	892	187	133

### Clinical data management

A central ATiM database was created and customized for the CPCBN repository in which clinical data were entered and yearly updated using a standardized process. The database was subjected to quality control measures to assess the degree of entry error or missing information across all centers (Fig. 1, left panel). An audit was performed on entries for 10% of patients contributed by each site. Based on this exercise, 6 out of 6309 data entries resulted in an error rate of 0.09%, which was taken into account for standardization of data and future database updates (data not shown).

### Demographic of the CPCBN cohort

**Fig. 4:**
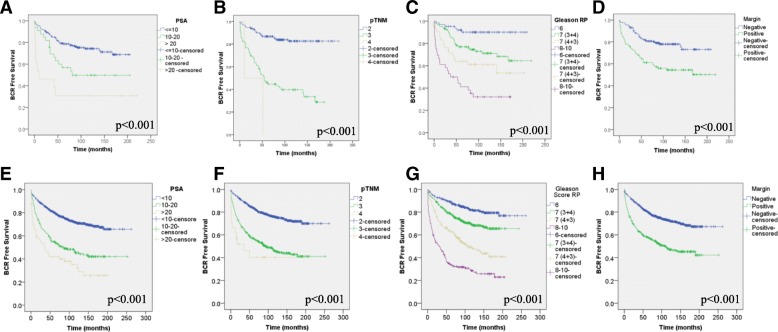
Kaplan-Meier plots showing relationship of clinical parameters with biochemical relapse (BCR). Both cohort of patients, Test (**a-d**) and Validation (**e-h**), were assessed independently. Clinical parameter evaluated were PSA level prior to surgery (**a, e**), pTNM (**b, f**), Gleason grade (**c, g**) and margin status (**d, h**). Statistical significance was set at *p* < 0.05

The median patient follow-up of patients was approximately 9.8 years, and a sufficient number of patients presented with endpoint elements such as BCR (34%), development of bone metastasis (4.3%) and death from PC (2.6%) to perform statistical analyses. Details on the clinico-pathological data of cohorts of patients whose prostate tissues were included in the Test-TMA as well as the Validation-TMA are presented in Table 4. In order to determine if the CPCBN cohort was representative of a general PC cohort, Cox regression analyses and Kaplan-Meier curves coupled with log-rank tests were performed using clinical parameters known to be associated with patient prognosis. As expected, PSA serum levels prior to surgery, pathological TNM, Gleason grade and margin status showed an association with BCR in both Test and Validation cohorts (Table 5 and Fig. 4). All clinical parameters except for margin status were also associated with the development of bone metastasis (Table 5 and Additional file 3: A-H) and death (Table 5 and Additional file 3: I-L).

**Table 4:** Clinico-pathological features of prostate cancer patients treated by radical prostatectomy

TMA series		Test		Validation	
Number of patients		250		1262	
Mean age at diagnosis		61		61	
Median follow-up (months)		113		120	
		N	%	N	%
Gleason score at RP	≤3 + 3	64	25.6	392	31.1
	3 + 4	104	41.6	499	39.5
	4 + 3	42	16.8	188	14.9
	≥4 + 4	36	14.4	175	13.9
	NA	4	1.6	8	0.6
pTNM	2	171	68.4	788	62.4
	3	77	30.8	453	35.9
	4	2	0.8	21	1.7
Margin status	Negative	156	62.4	837	66.3
	Positive	91	36.4	418	33.1
	NA	3	1.2	7	0.6
Biochemical relapse	No	173	69.2	828	65.6
	Yes	77	30.8	434	34.4
Type of biochemical relapse	Rising PSA	54	21.6	264	20.9
	Failed RP	16	6.4	85	6.7
	Treatment	7	2.8	85	6.7
Bone metastasis	No	239	95.6	1208	95.7
	Yes	11	4.4	54	4.3
Castrate resistant	No	237	94.8	1201	95.2
	Yes	13	5.2	61	4.8
Mortality	PC specific	4	1.6	36	2.9
	Other cause(s)	17	6.8	119	9.4
	Overall	21	8.4	155	12.3

*TMA* tissue microarray, *RP* radical prostatectomy, *pTNM* pathological staging, *NA* not available, *Rising PSA* serum level of prostate-specific antigen (PSA) of 0.2 ng/mL and rising, *Failed RP* PSA level after surgery > 0.2 ng/mL, *PC* prostate cancer

**Table 5:** Cox regression analyses of clinico-pathological parameters on the Test- and Validation-TMA cohorts

Endpoint	Clinical parameter	Test-TMA cohort				Validation-TMA cohort			
		*P*	Exp(B)	95.0% CI		*P*	Exp(B)	95.0% CI	
				Lower	Upper			Lower	Upper
BCR	Serum PSA level	**< 0.001**	**1.064**	**1.042**	**1.086**	**< 0.001**	**1.031**	**1.026**	**1.036**
	Gleason score at RP (6, 3 + 4, 4 + 3, > 8)	**< 0.001**	**2.035**	**1.631**	**2.54**	**< 0.001**	**1.946**	**1.778**	**2.13**
	pTNM	**< 0.001**	**4.673**	**3.133**	**6.97**	**< 0.001**	**2.599**	**2.202**	**3.067**
	Margin status	**< 0.001**	**2.392**	**1.517**	**3.77**	**< 0.001**	**2.362**	**1.955**	**2.852**
Bone metastasis	Serum PSA level	**0.051**	**1.047**	**1**	**1.096**	**0.047**	**1.018**	**1**	**1.036**
	Gleason score at RP (6, 3 + 4, 4 + 3, > 8)	**0.001**	**3.159**	**1.6**	**6.237**	**< 0.001**	**3.333**	**2.476**	**4.487**
	pTNM	**< 0.001**	**8.396**	**3.043**	**23.162**	**< 0.001**	**3.882**	**2.422**	**6.22**
	Margin status	0.125	2.624	0.765	9.008	0.988	0.996	0.569	1.743
PC specific death	Serum PSA level	–	–	–	–	**0.046**	**1.02**	**1**	**1.039**
	Gleason score at RP (6, 3 + 4, 4 + 3, > 8)	**0.001**	**3.159**	**1.6**	**6.237**	**< 0.001**	**3.333**	**2.476**	**4.487**
	pTNM	–	–	–	–	**< 0.001**	**3.263**	**1.843**	**5.78**
	Margin status	–	–	–	–	0.117	1.689	0.877	0.3252

*TMA* tissue microarray, *95% CI* 95% confidence interval, *BCR* biochemical recurrence, *PSA* prostate-specific antigen, *RP* radical prostatectomy, *pTNM* pathological staging, *PC* prostate cancer. Bold indicate significance

## Discussion

The mandate of the CPCBN is to identify the best set of molecular markers that will complement current parameters for clinical decision-making in PC. The underlying incentive behind this pursuit is to minimize adverse health complications that result from overtreatment of clinically-indolent PC. Current diagnostics are unable to resolve the range of heterogeneity and individualized risk of patients. Although active surveillance is now an option, still too many newly diagnosed patients with early-stage tumors are aggressively treated to safeguard them against the potential fraction of tumors that progress or cause lethal disease. Despite several reports of proposed biomarkers with prognostic impact, most have been reported in the context of small cohorts, same-institution studies, or lack follow-up patient data, introducing a level of bias that limits their validation for broad clinical use [3, 5, 11, 12]. The CPCBN addresses the outstanding need for validating existing biomarkers with a TMA-based platform to validate tissue markers. With large cohorts of adequate power, standardized protocols, and extensive clinical information centralized into one database, the CPCBN validation platform provides a resource to refine a panel of markers that can be readily integrated into clinical practice.

The first phase of this initiative involved the construction of the QC-TMA, which demonstrated the quality and feasibility of a multi-center TMA resource. The results of this exercise provided a logistical assurance in building large-scale cohorts from five participating biobanks, without site-specific bias. More noteworthy was the overall informative quality of cores that were evaluated by IHC and FISH techniques already used in clinical pathology practice and diagnostic labs. With H&E staining and reference markers of tissue integrity and malignancy, we were able to assess that 94% of samples provided at least two informative cores of high quality. FISH results also demonstrated a distribution of *PTEN* deletions among tumor cores that were aligned with previous reports in the literature [13]. With a homogeneous patient cohort, we were able to accumulate a large sample size (*n* = 1512) based on power calculations that would bestow statistical significance on biomarker performance. Division of the RP cohort into a Test-TMA (*n* = 250) and Validation-TMA (*n* = 1262) enhances the selection process and power of the platform, centered upon a rigorous checkpoint scheme in which biomarker status is assessed at several stages with a “Go or No-Go” decision tree. With the addition of an OPT-TMA to ensure the efficacy of conditions and staining protocols with prospective candidates, the sequence of testing from OPT-TMA to Test-TMA to Validation-TMA guards against wasting efforts with weak biomarkers, and preserving the Validation-TMA for the most robust candidates.

CTRNet policies and SOPs, patient information is updated each year on the ATiM database. In addition updates regarding the progress status of biomarkers and associated TMAs that are assigned to each project are also entered into the database. The centralized database can also coordinate several projects for meta-analysis and help to develop nomograms that will combine current parameters with biomarker analyses and integrate age, co-morbidity, clinico-pathological staging to ultimately define an accurate profile indicating individual risk for each PC patients. The application of emerging nomograms including biomarkers could be useful in the decision-making process with correlative evidence-based science to guide patient care.

## Conclusions

In conclusion, the CPCBN RP TMA has been constructed, controlled for quality and is available, in a step-wise manner, for researchers who intend to validate prognostic biomarkers in prostate cancer (for more information see http://www.tfri.ca/en/research/translational-research/cpcbn/cpcbn_access.aspx). As the first completed TMA series of the CPCBN-TMA platform, this RP cohort will serve as a prototype model that will facilitate the assembly of future retrospective and prospective cohorts for biomarker validation. Altogether the CPCBN-TMA platform will serve as an invaluable resource for the entire PC research community, accelerating breakthroughs in PC research, and supporting the establishment of nomograms to predict progression.

## Additional files

Additional file 1: Antibodies and conditions for automated immunohistochemistry staining. This table contained the antibodies and conditions for immunohistochemistry staining. (DOCX 13 kb)

Additional file 2: Core scores of QC-TMA based on FISH analysis of *PTEN* deletion status. This table contains information regarding the core quality for FISH scoring. (DOCX 12 kb)

Additional file 3: Kaplan-Meier plots showing relationship of clinical parameters with patient outcome. Both cohort of patients, Test (A-D) and Validation (E-L), were assessed independently for the endpoints of bone metastasis (A-H) and prostate cancer-specific mortality (I-L). Clinical parameter evaluated were PSA level prior to surgery (A, E), pTNM (B, F), Gleason grade (C, G) and margin status (D, H). Statistical significance was set at *p* < 0.05. (PPTX 302 kb)

## Abbreviations

ASAP: Atypical small acinar proliferation
ATiM: Advanced Tissue Management
BCR: Biochemical relapse
CHUdeQ-UL: Centre hospitalier universitaire de Québec-Université Laval CHUdeQ-UL
CHUM: Centre hospitalier de l’Université de Montréal
CPCBN: Canadian prostate cancer biomarker network
CTRNet: Canadian Tumor Repository Network
FFPE: Formalin-fixed paraffin-embedded
FISH: Fluorescence in situ hybridization
GS: Gleason score
H&E: Hematoxylin and eosin
IDC: Intraductal carcinoma
IHC: Immunohistochemistry
IRB: Institutional Review Board
MUHC: McGill University Health Centre
OPT-TMA: Optimization TMA
PC: Prostate cancer
PIN: Prostatic intraepithelial neoplasia
PSA: Prostate-specific antigen
QC-TMA: Quality control TMA
RP: Radical prostatectomy
SOPs: Standard operating procedures
TMA: Tissue microarray
UHN: University Health Network
VPC: Vancouver Prostate Centre
