# Supplementary material for: The Terry Fox Research Institute Canadian Prostate Cancer Biomarker Network: an analysis of a pan-Canadian multi-center cohort for biomarker validation
Source: BMC Urol. 2018 Sep 10;18:78. doi: 10.1186/s12894-018-0392-x (PMC6131811; doi:10.1186/s12894-018-0392-x)
Supplement: Supplementary file 1 — Antibodies and conditions for automated immunohistochemistry staining. This table contained the antibodies and conditions for immunohistochemistry staining. (DOCX 13 kb) [file 12894_2018_392_MOESM1_ESM.docx]

**Additional File 1. Antibodies and conditions for automated immunohistochemistry staining**

| Antibody | Company | Cat. Number | Clone | Ventana staining | | |
| --- | --- | --- | --- | --- | --- | --- |
|  |  |  |  | Antigen Retrieval CC1 | Antibody | |
|  |  |  |  | Time (min) | Dilution | Time (min) |
| p63 | Biocare Medical | PM 163 AAK | BC4A4 | 36 | 1:200 | 32 |
| p504s | DAKO | GA06061-2 | 13H4 | 36 | 1:200 | 32 |
| p501s | DAKO | M361529-2 | 10E3 | 36 | 1:200 | 32 |
| PSMA | DAKO | M362029-2 | 3E6 | 72 | 1:200 | 32 |
| CK18 | Santa Cruz | sc-6259 | DC-10 | 30 | 1:2000 | 40 |
| HMW-CK | Cedarlane | CLSG36689-05 | 34ßE12 | 60 | 1:100 | 40 |
| AR | Diagnostic Biosystems | Mob 245-05 | AR-441 | 60 | 1:100 | 60 |
| Ki67 | Thermo Fisher | RM-9106 | SP6 | 60 | 1:1000 | 44 |
| PSA | DAKO | A0562 | - | 60 | 1:100 | 60 |

CC1: Cell Conditioning 1 solution, supplied by Ventana Medical Systems, Inc.
