# Supplementary material for: The Terry Fox Research Institute Canadian Prostate Cancer Biomarker Network: an analysis of a pan-Canadian multi-center cohort for biomarker validation
Source: BMC Urol. 2018 Sep 10;18:78. doi: 10.1186/s12894-018-0392-x (PMC6131811; doi:10.1186/s12894-018-0392-x)
Supplement: Supplementary file 2 — Core scores of QC-TMA based on FISH analysis of PTEN deletion status. This table contains information regarding the core quality for FISH scoring. (DOCX 12 kb) [file 12894_2018_392_MOESM2_ESM.docx]

**Additional File 2. Core scores of QC-TMA based on FISH analysis of *PTEN* deletion status**

| Number of cores | Very Good | Intermediate | Poor |
| --- | --- | --- | --- |
| CHUdeQ-UL | 5 | 13 | 12 |
| CHUM | 7 | 21 | 2 |
| MUHC | 13 | 14 | 3 |
| UHN | 14 | 13 | 3 |
| VPC | 12 | 18 | 0 |
| Total | 51 | 79 | 20 |
|  |  |  |  |
| Percent of cores | Very Good | Intermediate | Poor |
| CHUdeQ-UL | 17 | 43 | 40 |
| CHUM | 23 | 70 | 7 |
| MUHC | 43 | 47 | 10 |
| UHN | 47 | 43 | 10 |
| VPC | 40 | 60 | 0 |
| Total (%) | 34 | 53 | 13 |
