# Supplementary figures and images for: The Terry Fox Research Institute Canadian Prostate Cancer Biomarker Network: an analysis of a pan-Canadian multi-center cohort for biomarker validation
Source: BMC Urol. 2018 Sep 10;18:78. doi: 10.1186/s12894-018-0392-x (PMC6131811; doi:10.1186/s12894-018-0392-x)

## Slide 1
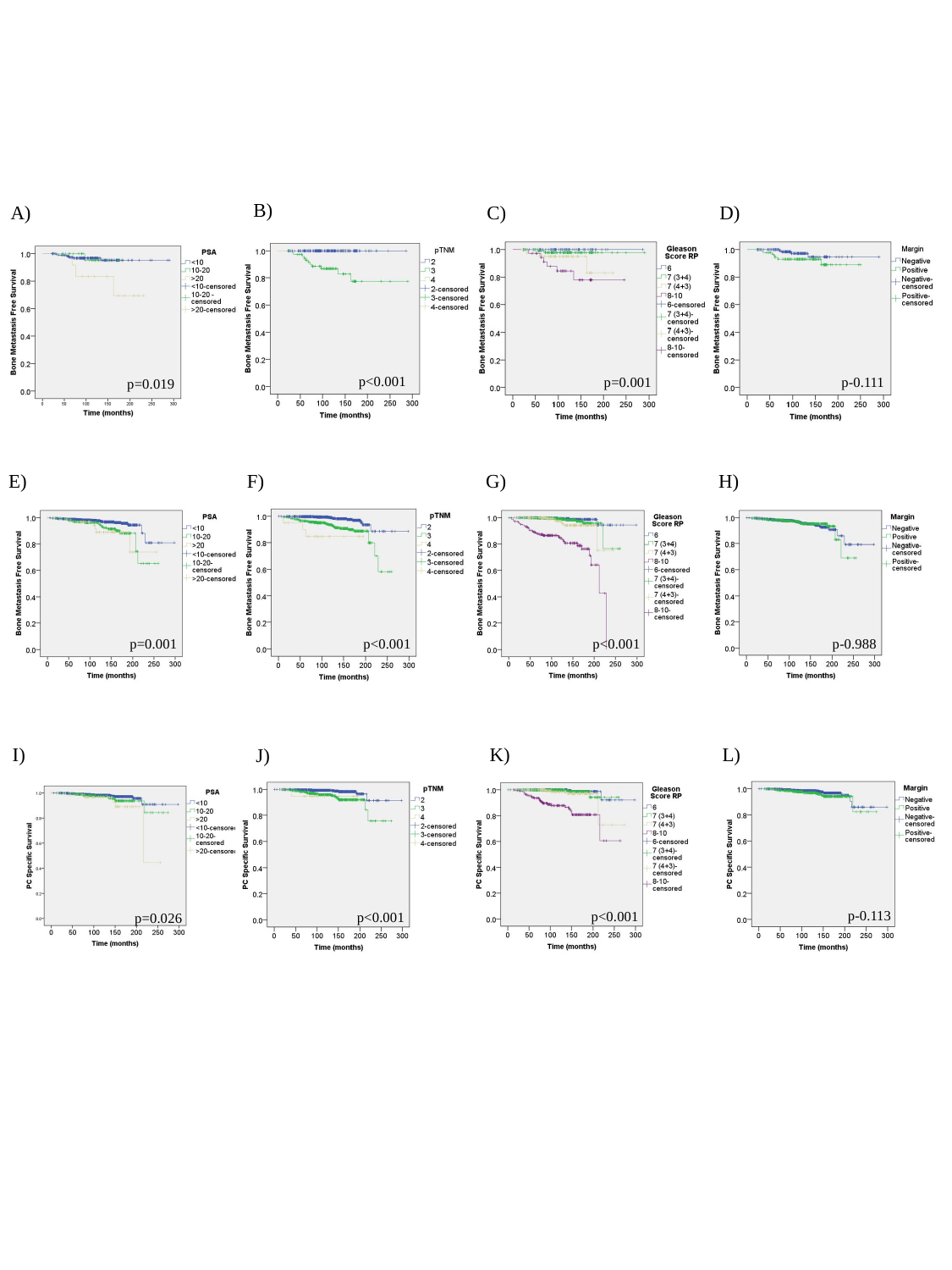

B)
A)
C)
D)
p<0.001
p=0.001
p-0.111
p=0.019
E)
F)
G)
H)
p-0.988
p=0.001
p<0.001
p<0.001
I)
K)
L)
J)
p-0.113
p<0.001
p<0.001
p=0.026

Supplement: Supplementary file 3 — Kaplan-Meier plots showing relationship of clinical parameters with patient outcome. Both cohort of patients, Test (A-D) and Validation (E-L), were assessed independently for the endpoints of bone metastasis (A-H) and prostate cancer-specific mortality (I-L). Clinical parameter evaluated were PSA level prior to surgery (A, E), pTNM (B, F), Gleason grade (C, G) and margin status (D, H). Statistical significance was set at p < 0.05. (PPTX 302 kb) [file 12894_2018_392_MOESM3_ESM.pptx]
